# Subcortical evoked activity and motor enhancement in Parkinson's disease

**DOI:** 10.1016/j.expneurol.2015.12.004

**Published:** 2016-03

**Authors:** Anam Anzak, Huiling Tan, Alek Pogosyan, Sadaquate Khan, Shazia Javed, Steven S. Gill, Keyoumars Ashkan, Harith Akram, Thomas Foltynie, Patricia Limousin, Ludvic Zrinzo, Alexander L. Green, Tipu Aziz, Peter Brown

**Affiliations:** a Functional Neurosurgery–Experimental Neurology Group, Nuffield Department of Clinical Neurosciences, University of Oxford, Level 6, West Wing, John Radcliffe Hospital, OX3 9DU, United Kingdom; b Sobell Department of Motor Neuroscience & Movement Disorders, UCL Institute of Neurology, United Kingdom; c Nuffield Department of Clinical Neurosciences, University of Oxford, Level 6, West Wing, John Radcliffe Hospital, OX3 9DU, United Kingdom; d Department of Neurosurgery, Institute of Neurosciences, Frenchay Hospital, Bristol, United Kingdom; e Department of Neurosurgery, King's College Hospital, Kings College London, United Kingdom

**Keywords:** Subthalamic nucleus, Pedunculopontine nucleus, Arousal, Evoked activity, Local field potentials

## Abstract

Enhancements in motor performance have been demonstrated in response to intense stimuli both in healthy subjects and in the form of ‘paradoxical kinesis’ in patients with Parkinson's disease. Here we identify a mid-latency evoked potential in local field potential recordings from the region of the subthalamic nucleus, which scales in amplitude with both the intensity of the stimulus delivered and corresponding enhancements in biomechanical measures of maximal handgrips, independent of the dopaminergic state of our subjects with Parkinson's disease. Recordings of a similar evoked potential in the related pedunculopontine nucleus – a key component of the reticular activating system – provide support for this neural signature in the subthalmic nucleus being a novel correlate of ascending arousal, propagated from the reticular activating system to exert an ‘energizing’ influence on motor circuitry. Future manipulation of this system linking arousal and motor performance may provide a novel approach for the non-dopaminergic enhancement of motor performance in patients with hypokinetic disorders such as Parkinson's disease.

## Highlights

• We identify an evoked potential in the subthalamic nucleus of Parkinsonian patients.
• This scales with enhancements in motor performance by loud sounds.
• The same is seen in the pedunculopontine nucleus of the reticular activating system.
• The potential may be a neural marker of movement energization by phasic arousal.

## 1 Introduction

A brief enhancement of motor performance in response to intense, alerting, or arousing stimuli, is a commonly experienced phenomenon. Under such circumstances, experimental evidence has shown that even *peak* motor responses can undergo augmentation, over and above the effects of maximal effort of will, both in healthy subjects (Woodworth, 1938, Angel, 1973, Anzak et al., 2011a) and in patients ordinarily hindered by the bradykinetic symptoms of Parkinson's disease (PD) (Valldeoriola et al., 1998, Ballanger et al., 2006, Anzak et al., 2011b, Anzak et al., 2012). Anecdotal reports of a comparable effect – termed ‘paradoxical kinesis’ (Souques, 1921) – have described patients with advanced PD being able to jump up and run at the sound of a car accident (Daroff, 2008), the sensation of an earthquake (Bonanni et al., 2010), or sight of an approaching wall of flood-water (Schwab and Zieper, 1965). However, the neural pathways driving this remarkable phenomenon have remained enigmatic.

Accordingly, in the current study we investigate the possible mechanisms underlying the augmentation of peak motor performance by arousing stimuli. As a number of studies have now implicated a role of the basal ganglia in the scaling of voluntary movement (Turner et al., 2003, Thobois et al., 2007, Muthukumaraswamy, 2010, Grafton and Tunik, 2011, Brücke et al., 2012, Tan et al., 2015) including that at maximal effort (Anzak et al., 2012, Joundi et al., 2012), we test the hypothesis that activity in this network responsible for movement gain (Turner and Desmurget, 2010) also helps mediate additional enhancements in motor performance with arousing stimuli. To this end we capitalize on the unique opportunity afforded by therapeutic deep brain stimulation to record from key subcortical nuclei.

## 2 Materials and methods

### 2.1 Subjects

All subjects gave their informed consent to take part in the study, which was approved by the local ethics committees at our recording sites in Oxford, London and Bristol, United Kingdom. Eight patients with PD (mean disease duration 11.6 years, mean age 57.1 years, range 32–70 years, six males) underwent bilateral implantation of DBS electrodes into the STN, as a prelude to therapeutic high frequency stimulation for advanced idiopathic PD with motor fluctuations and/or dyskinesias. Techniques to target and implant electrodes in the STN have previously been described (Foltynie and Hariz, 2010). No microelectrode recordings were made, although the effects of direct stimulation were confirmed intra-operatively. In addition, the locations of the electrodes were confirmed with immediate post-operative stereotactic imaging. Nonetheless, in acknowledgment of the fact that not all electrode contacts could be expected to lie in the STN per se, we term the area sampled by the contact pairs the STN region, STNr. DBS electrode extension cables were externalized through the scalp to enable recordings prior to connection to a subcutaneous DBS pacemaker, implanted in a second operative procedure up to seven days later. Clinical details of the patients are available in Table 1. The mean percentage improvement in the motor section of the Unified Parkinson's Disease Rating Scale (UPDRS) on treatment with levodopa (L-DOPA) was 70.0 ± 5.6% (p = 0.018, Wilcoxon signed-rank test between ON and OFF L-DOPA scores; data missing in one case) across subjects, indicating good responsiveness to L-DOPA in our study participants. A further PD patient was implanted in the pedunculopontine region (PPNr) and STNr/zona incerta, bilaterally, for freezing of gait (see last case in Table 1). The PPNr electrodes were placed using a transventricular trajectory so that all four electrode contacts were intended to lie within the PPN. The STNr electrodes were placed in the caudal zona incerta, with the central two electrode contacts lying adjacent to or clipping the STN. Details of the surgical procedure have been previously outlined (Khan et al., 2011).

### 2.2 Experimental paradigm

Subjects were presented with a series of imperative visual (V) cues, separated by 8.0 ± 0.5 s, and instructed to squeeze a hand-held force dynamometer “as fast and hard as you possibly can when the light comes on and maintain this for the duration of the light” (red light-emitting-diode illuminated for 3 s). A loud auditory stimulus (40 ms duration, 1 kHz), at one of five different randomly selected sound pressure levels (82, 88, 94, 100, 105 dB) was delivered binaurally through headphones, with onset simultaneous with that of the V cue. However, subjects were asked to just focus on responding to the V cues. Fifteen cues of each sound pressure level (75 trials in total) were delivered in each experimental run. Trials were carried out in a blocked design, and left- and right-hand recordings were counterbalanced across subjects. Inter-trial intervals were shorter than in our previous studies (Anzak et al., 2011a, Anzak et al., 2011b, Anzak et al., 2012) to allow for a greater number of trials to be executed prior to correlative analysis, whilst avoiding an excessively lengthy paradigm in our patients with PD.

Grip force was measured one hand at a time in each subject using an isometric dynamometer (G100; Biometrics Ltd, Cwmfelinfach, Gwent, UK), with standard Jamar design and its handle set in the second of the five discrete grip diameter adjustments possible (Sancho-Bru et al., 2008). Subjects were seated with their shoulders adducted (so that elbows rested against the trunk), their elbows flexed at about 90° and their forearms in neutral, as recommended by the American Association of Hand Therapists (Fess, 1992). Stimulus intensities were measured in a sound-proofed room with a Brüel and Kjaer 2260 Observer (Brüel and Kjaer, Nærum, Denmark) via an artificial ear and headphone adapter.

### 2.3 Recordings

In our nine patients with externalized DBS electrodes (8 bilateral STNr, 1 bilateral PPNr & STNr/ZI), LFP recordings were made 3–6 days after surgery. In order to complete the recordings in one morning, and limit intrusion on our easily fatigable post-operative patients, recordings were always made first after overnight withdrawal of anti-parkinsonian medication (OFF L-DOPA), and then again approximately 1 h after taking their usual morning dose (average morning L-DOPA dose administered = 186 ± 62 mg). Improvement with medication was confirmed through assessment of finger tapping, wrist rigidity and tremor (using the corresponding items of the motor UPDRS).

LFPs, and surface EEG from Fz and Cz were recorded monopolarly with respect to a linked earlobe reference using a TMSi porti amplifier (TMS international) and its respective software. EMG was recorded from orbicularis oculi to identify blinks. All recordings were band-pass filtered between 0.5 and 500 Hz and sampled at 2048 Hz. Analogue correlates of the visual and auditory stimuli and dynamometer output were recorded and digitized in a similar way. Monopolar LFP recordings were subsequently converted off-line to a bipolar montage between adjacent contacts (three bipolar channels per side) to limit the effects of volume conduction from distant sources. Bipolar Fz–Cz was also created offline. The line noise artefacts at 50 Hz and 100 Hz were removed using notch filters (5th order zero-phase Butterworth filters).

### 2.4 Data analysis

Analyses of both behavioral and LFP data were performed in Matlab (version 7.10). Peak force (PF) and pre-motor reaction time (RT) were the chosen biomechanical variables of interest. Premotor reaction time was defined as the time interval between cue onset and the point at which force exceeded 5% of the PF (taken as response onset). We acknowledge that premotor reaction time is more usually considered to be the interval between cue presentation and EMG onset (Botwinick and Thompson, 1966). However, as in previous studies (Anzak et al., 2011a, Anzak et al., 2011b, Anzak et al., 2012) we found the use of EMG to be suboptimal in the context of maximal grips because of movement artefact and sampling error, due to activation of multiple muscles in this task. Peak yank (PY; where yank is defined as the rate of change of force, calculated by differentiation of the force signal) was also derived. Note too that the scalp EEG was contaminated by auditory blink reflexes to the intense stimuli and so was not analyzed further.

STNr LFP activity recorded from externalized DBS electrodes was decomposed into two components: evoked potentials, which are phase-locked to stimulus onset, and induced frequency-specific components, which are not (David et al., 2006). We sought to derive both these constituents in order to investigate whether STNr activity in either domain preceded and correlated with enhancements in motor performance. The techniques for derivation of evoked activity and event related induced power are outlined in Supplementary Material 1.

### 2.5 Statistics

Grand averages of PF, PY and RT, in response to each stimulus intensity, were calculated after deriving each of these variables from the individual grips made by a subject, and calculating the mean values for that subject, before averaging across study participants.

**Fig. 1:**
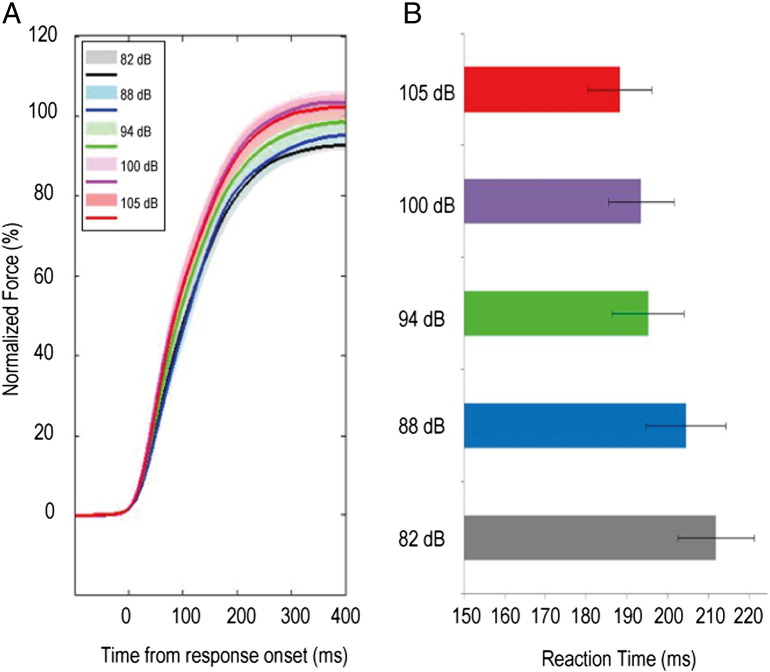
Normalized group average (A) grip force (B) reaction times, in response to five different cue intensities averaged across OFF and ON L-DOPA recordings. n = 16 gripping hands. Traces in (A) have been normalized as a percentage of the maximal peak force attained among different trials by an individual in response to the maximum intensity stimulus, before collation across subjects. Shaded area corresponding to each cue intensity represents the 95% confidence interval (CI) of the force traces. Note, where the CIs overlap, the CI of the greater amplitude trace is shown. Group mean RTs are shown with SEM.

For calculation of Pearson's correlation coefficients and multiple linear regression analyses at the within-subject level, behavioural and LFP data – in response to each stimulus intensity – were normalized as a proportion of responses to the lowest intensity (82 dB) stimulus, with exception of the data illustrated in Fig. 1. In order to achieve a more uniform spread of the data for all parametric analyses, behavioral parameters and peak evoked potential amplitudes were log transformed, whilst mean induced power over specific frequency bands benefited the most from a square root transform of the absolute amplitude of the activity (preserving the polarity of the data). High beta activity in one individual was excluded from correlative analyses, as the average power was eight times the greatest high beta power from among the remaining individuals.

In illustrations of evoked activity, the time-evolving power of the group mean absolute evoked potential traces, for each stimulus intensity, has been plotted as a z-score calculated for the average evoked potential profiles derived from all contact pairs on each DBS electrode in an individual, before collapsing across subjects. Z-scores were derived from the power of the absolute amplitude time series of the evoked potential at each millisecond interval by subtraction of the mean LFP power 1–2 s prior to the cue, followed by division of this value by the standard deviation of the power of the 1–2 s pre-cue baseline. These illustrations therefore exclude induced power.

Statistical analyses were performed in SPSS Statistics 19 (SPSS Inc., Chicago, IL, USA). Kolmogorov-Smirnov tests were applied for confirmation of normal distributions, where required, prior to parametric testing. Where Mauchly's test of sphericity was significant (p < 0.05) in repeated measures ANOVAs, Greenhouse–Geisser corrections were applied. Mean ± standard error of mean (SEM) are presented throughout the text. Those statistical tests that reached significance (p < 0.05), and where appropriate survived correction for multiple comparisons using the Bonferroni correction (Curran-Everett, 2000) are indicated with an asterisk (*) in the text.

## 3 Results

### 3.1 Progressive enhancements in peak motor performance with increasing stimulus intensity in patients and healthy controls

Group average RTs and the average shape of the maximal grip profile in patients (n = 16 gripping hands), with increasing stimulus intensity, are shown in Fig. 1. PF increased with stimulus intensity, with the exception that the response to the highest two sound intensities was indistinguishable. RTs decreased in a monotonic fashion with increasing stimulus intensity. Application of repeated measures ANOVAs to PF and RT data confirmed significant (p < 0.05) effects of stimulus intensity on each parameter (see Table 2). The absence of an interaction of stimulus intensity with medication, confirmed an independence of the motor enhancements observed from dopaminergic manipulations (3,6,7). Note also in Table 2, group average reduction in PF with dopaminergic medication (p = 0.035*, 16.7 ± 1.6 kg ON medication as compared to 19.4 ± 2.0 kg OFF medication, when averaging across responses to all stimulus intensities) likely reflects greater fatigue in ON drug recordings as compared to OFF, as the latter were necessarily conducted prior to the former (see Materials and methods). Group mean PF and RT averaged across dopaminergic states changed from 17.7 ± 1.8 kg, and 211.9 ± 9.4 ms in response to the lowest intensity stimulus, to 18.5 ± 1.7 kg, and 188.3 ± 7.9 ms in response to the highest intensity stimulus. In six healthy, age-matched controls, significant increases in PF and reductions in RT were similarly observed (Supplementary Fig. 1, Supplementary Table 1). Group mean PF and RT averaged across experimental runs changed from 13.1 ± 1.2 kg, and 164.6 ± 6.4 ms in response to the lowest intensity stimulus, to 15.1 ± 1.5 kg, and 141.6 ± 5.1 ms in response to the highest intensity stimulus in the healthy subjects. There was no experimental run × stimulus intensity interaction, and this, together with the absence of a dopaminergic medication × stimulus intensity interaction in the patient group, allowed derivation of a mean response to stimulus intensity for each subject group, averaging across dopaminergic state/experimental run. Subsequent repeated measures ANOVAs with factors GROUP (patient vs healthy subject) and stimulus intensity (5 levels), revealed no significant group × stimulus intensity interactions, suggesting similarly proportional enhancements in motor performance of both groups to increasing stimulus intensity (Supplementary Table 2). The results pertaining to PY were broadly similar to those with PF in both subject groups, as demonstrated in Supplementary Fig. 2a, b and Supplementary Material 2.

### 3.2 A mid-latency evoked potential, with focal origin in the STNr, increases in amplitude with increasing stimulus intensity

**Fig. 2:**
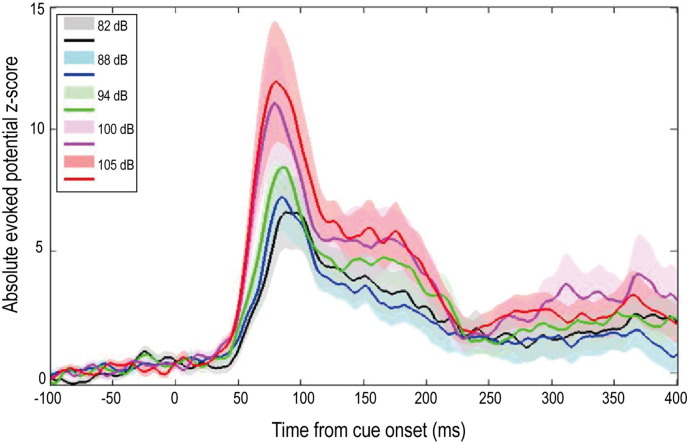
Group average STNr mid-latency evoked potentials in response to five different cue intensities, averaged across OFF and ON L-DOPA recordings. n = 16 STNr. Shaded area corresponding to each cue intensity represents the 95% CI of the evoked potential, and where the CIs overlap, the CI of the greater amplitude trace is shown. The absolute amplitude of the evoked potential is shown as a z-score (see Materials and methods).

Analysis of STNr LFP activity revealed an evoked potential which scaled in amplitude with stimulus intensity (Fig. 2). Repeated measures ANOVAs applied to mean peak evoked potential amplitudes (averaged across all contact pairs on each DBS electrode, and derived separately for OFF and ON L-DOPA recordings), with factors stimulus intensity (5 levels) and hemisphere (left and right STNr DBS electrodes), revealed main effects of stimulus intensity, but no effects of STNr side, nor STNr side × stimulus intensity interactions in either OFF or ON L-DOPA states (see Table 3). Importantly, a further repeated measures ANOVA applied to the average of the *bilateral* evoked potentials for each stimulus intensity identified an independence of the peak amplitude from dopaminergic state (significant effect of stimulus intensity, F_4,60_ = 9.390, p < 0.001*; but no effect of dopaminergic medication, F_1,15_ < 0.001, p = 0.999, nor stimulus intensity × dopaminergic medication interaction, F_4,60_ = 0.410, p = 0.800).

Averaging across recordings from all contact pairs in left and right sided DBS electrodes, and across OFF and ON L-DOPA experimental runs, the mean onset latency of this evoked potential in response to all stimulus intensities – defined here as the latency of a z-score (see Materials and methods) > 1 – was 42.4 ± 2.7 ms. Peak latencies fell between 50–100 ms, with mean peak latency (averaged across L-DOPA states, left and right sided DBS electrode contact pairs, and all stimulus intensities) falling at 83.3 ± 1.2 ms. Of note, the imperative visual cue alone resulted in an evoked potential with a peak latency of > 100 ms (see Supplementary Fig. 3). In line with its proposed role in arousal related processing, rather than in the direct control of movement per se (see Supplementary Fig. 4), a paired t-test between evoked potential amplitude in a ‘grip’ versus ‘no grip’ condition identified no significant difference (p = 0.087) in the peak evoked amplitudes recorded. However, this in-of-itself would not preclude an influence of the evoked potential on voluntary movement when the latter is made.

In the current study, local generation of the evoked potential was confirmed by the steep gradient in the peak amplitude of the evoked potential between the contact pair in which it was maximal and the remaining contact pairs (56.0 ± 2.1% drop averaged across responses to all sound intensities in 8 subjects). The contact pair recording the greatest amplitude evoked potential was 0–1 in 39% of cases, 1–2 in 31% of cases and 2–3 in 30% of cases. In a further sub-analysis of responses to the highest intensity stimulus, the evoked potential co-localized with the contact pair recording the greatest amplitude broad gamma (34–375 Hz up to 0.5 s from cue onset) in 83.3% of the cases, in line with an origin in the sensorimotor STN (Trottenberg et al., 2006). Examples of polarity reversal of the evoked potential in those contact pairs adjacent to the pair recording the greatest amplitude evoked potential (see Supplementary Fig. 5) provide further support for a focal origin of the potential.

### 3.3 Induced frequency-specific components of LFP activity are not associated with increases in stimulus intensity

Derivation of time-evolving power spectra of changes in STNr LFPs separately for both OFF and ON L-DOPA recordings, identified five frequency bands reactive to cue (see Supplementary Material 1; Supplementary Fig. 6). However, application of repeated measures ANOVAs to the average induced power retrieved from each frequency range identified an absence of an effect of stimulus intensity on any of the induced activities identified (Supplementary Table 3).

### 3.4 Enhancements in peak motor performance correlate with amplitude of the STNr mid-latency evoked potential

**Fig. 3:**
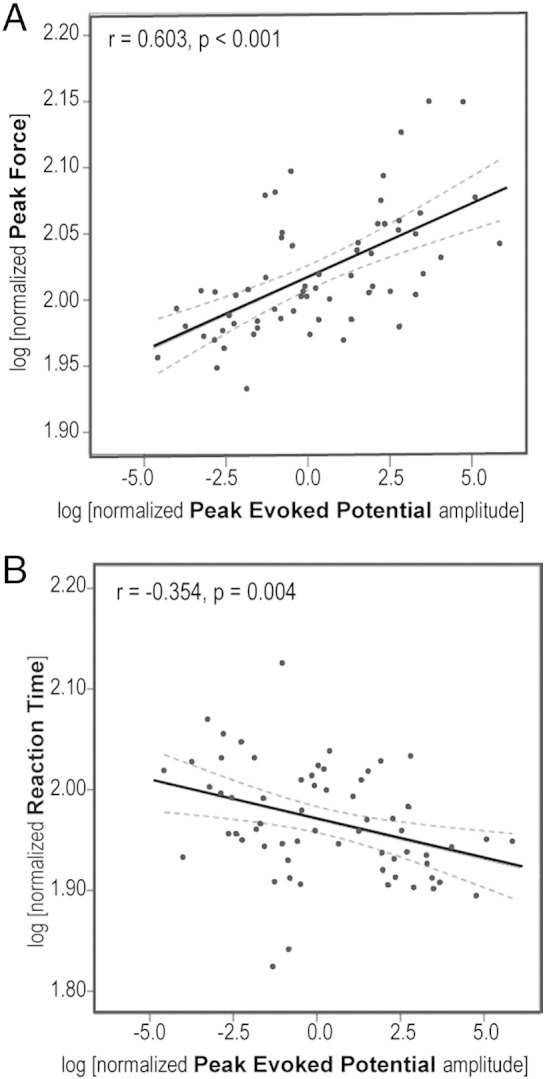
Scatter-plots relating increases in average absolute peak evoked potential amplitude to enhancements in (A) peak force and (B) reaction time, relative to the average of peak responses to the lowest intensity imperative cues. Data are derived from the remaining four sound intensities in the eight subjects. Solid lines represent best-fit lines fitted by simple linear regression. Dashed lines represent 95% CI of the regression line.

Potential relationships between the peak amplitude of the mid-latency evoked potential and motor enhancement were next investigated. Both the evoked potentials and movement parameters were normalized to the average response to the lowest stimulus intensity, so that relationships were investigated at the within-subject level, although correlation analysis was then confined to only 4 stimulus intensities. Simple regression analysis (applied to log transformed data) identified significant linear relationships between the peak amplitude of the STNr evoked potential and PF (r = 0.603, p < 0.001*) as well as RT (r = − 0.354, p = 0.004*) (Fig. 3). Of note, there was little evidence to suggest that evoked potential amplitude was a simple linear product of stimulus intensity (r = 0.140, p = 0.270; Supplementary Fig. 7). Similarly, as suggested by Fig. 1, a simple linear relationship between neither PF nor RT and stimulus intensity (r = 0.243, p = 0.053 and r = − 0.214, p = 0.090, respectively), was found.

In line with the absence of effect of stimulus intensity on induced frequency specific components of LFP activity, no significant correlations were found between these and the movement parameters of interest. Accordingly, a multiple regression model that included both peak evoked potential amplitude and transformed (see Methods) mean LFP power of induced components over the five identified frequency bands (ie. six predictive variables in total) revealed that only evoked potential amplitude, and not induced frequency-specific activities, contributed to PF (β = 0.654, p < 0.001*). The model fit was good: F = 7.902, p < 0.001*, R^2^ = 0.412. However, the fit was non-significant in a similar model applied to RT (F = 1.584, p = 0.170, R^2^ = 0.056), in agreement with the weaker correlations identified with simple regression analysis. No significant correlations between any of the induced frequency specific activities and the evoked potential amplitude were found.

Finally, peak yank demonstrated similar correlations with evoked activity (Supplemental Material 2; Supplementary Fig. 2).

### 3.5 An evoked potential, with similar latency and morphology to that in the STNr, is locally generated in the PPNr

**Fig. 4:**
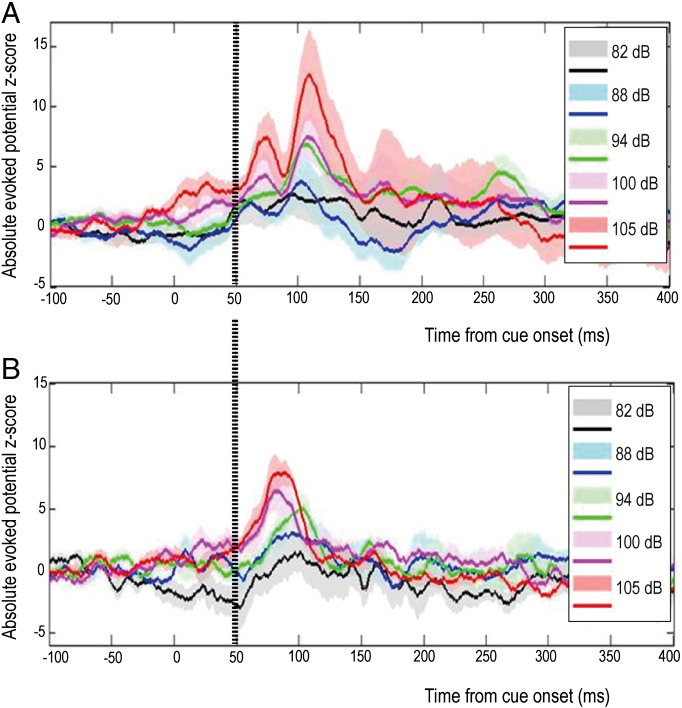
Average evoked potentials recorded simultaneously in (A) PPNr and (B) STNr. Traces represent the grand averages of recordings from left and right nuclei of a single patient whilst OFF and ON L-DOPA. Normalization technique and CI representation are as in Fig. 2. Dashed vertical line denotes the common onset latency of the first evoked potential in both structures.

**Fig. 5:**
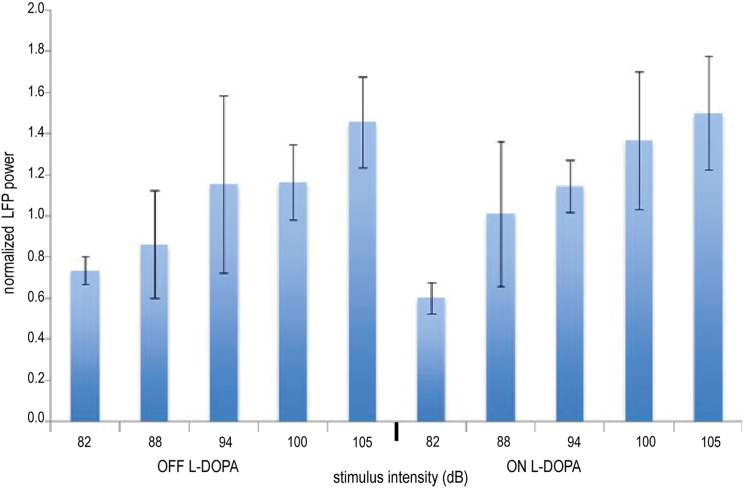
Average peak amplitude of the absolute evoked potentials recorded from the PPNr, in response to five different cue intensities. n = 2 PPNr, one individual. Average responses in experimental runs when the patient was OFF followed by ON their normal antiparkinsonian medication (L-DOPA) are shown. Error bars represent SEM of recordings averaged across all contact pairs in left and right PPN DBS electrodes. Evoked potential amplitudes were normalized to the greatest amplitude peak across the trials recorded in response to the lowest intensity stimulus, for each PPN, before averaging across sides.

We also recorded evoked activity in the PPNr during execution of the same gripping paradigm in a further patient with PD in whom DBS electrodes were implanted bilaterally in both the PPNr and STNr. In this subject, the PPNr LFP activity was characterized by two components, the first of which was of very similar onset latency to the initial evoked potential in the patient's STNr (see vertical line in Fig. 4). PPNr evoked potentials scaled with sound intensity, without dependence on dopaminergic state (Fig. 5). Importantly, the steep gradient in the peak amplitude of the evoked potential between the contact pair in which it was maximal and the remaining contact pairs indicated local generation of the evoked potentials in the PPNr (gradient 50.0 ± 3.2%) and STNr (gradient 52.3 ± 3.4%). The PPNr contact pair recording the maximal mid-latency evoked potential bilaterally was 1–2. The STNr contact pairs with the maximal mid-latency evoked potentials were 0–1 on the left and 1–2 on the right.

## 4 Discussion

Our study has two main findings. First, we identify an evoked potential with onset latency < 50 ms, locally generated in the STNr, which scales with increasing stimulus intensity and correlates with enhancements in peak force and peak yank as well as reductions in reaction time. Second, we provide evidence of an evoked potential with a striking similarity to that in the STNr, but with focal origin in the PPNr — a key component of the reticular activating system (RAS).

What might be the basis for the loud auditory stimulus evoked potential locally generated in the STNr? An involvement in the modulation of movement, rather than in specific auditory processing per se, seems likely given the pre-eminent role of the STN in motor control (Turner and Desmurget, 2010). It has long been posited that a cue not only instigates specific processing related to stimulus analysis, but also ‘automatic alertness’ or ‘phasic arousal’ (Posner, 2008, Sanders, 1983). Here the latter term is used to describe an evoked increment in vigilance – of short duration – that is dependent upon stimulus attributes like novelty and intensity. It has previously been suggested that such a mechanism speeds early processes of movement preparation and action selection (Hackley and Valle-Inclán, 1999, Hackley, 2009, Hackley et al., 2009), as well as increasing activation in primary motor areas (Jepma et al., 2009). Accordingly, we posit that the mid-latency sub-cortical potential identified in the present study represents an electrophysiological correlate of phasic arousal, and that its co-existence in the STNr and PPNr, which can be considered part of the reticular activating system, may plausibly relate to the interaction between such arousal and motoric processing.

Of note, the demonstration of a linear relationship between enhancements in motor performance and the amplitude of the STNr mid-latency evoked potential, but not with changes in any induced frequency-specific activities, provides support for the functional dissociation of these two LFP components. A role of *induced* gamma and high frequency LFP power in promoting movement has been widely reported (Foffani et al., 2003, Özkurt et al., 2011, Brücke et al., 2012, Jenkinson et al., 2015). However, we have previously shown that whilst induced STN activity over a broad gamma range constitutes a substantial factor in optimizing an individual's peak motor response at maximal effort of will, its contribution to performance increments over and above this is only slight, when an imperative visual cue is accompanied by a loud auditory tone on random trials (Anzak et al., 2012).

The recording of a locally generated evoked potential in the PPNr, with a similar latency and reactivity to that of the mid-latency STNr evoked potential, implicates the ponto-mesencephalic limb of the ascending arousal system (Moruzzi and Magoun, 1949, Jones, 2008) as a potential source of the evoked subthalamic activity related to phasic arousal following a stimulus. Indeed, on the basis of the strong reciprocal connections known to exist between the STN and PPN (Bevan and Bolam, 1995, Hammond et al., 1983, Mena-Segovia et al., 2004, Aravamuthan et al., 2007), a potential role for the STN as one *bridge* between arousal and motor circuitry is plausible. The PPN–STN pathway may thus constitute an additional movement gain (Turner and Desmurget, 2010) system, continuously up-dating the motor system on levels of arousal, and acting in parallel to those afferent pathways related to emotional (Schmidt et al., 2009) or incentive motivation (Pessiglione et al., 2007) processing which have also been shown to influence motor output.

In such a framework, the mid-latency STNr evoked potential may reflect the propagation of arousal to a motor structure from which an ‘energizing’ influence on motor cortico–subcortical loops can be exerted. The role of the basal ganglia in providing ‘motor energy’ (De Ajuriaguerra, 1975), and a related loss of this in PD, have been recurring themes in the literature (Wernicke, 1899, Wernicke, 1906, Trousseau, 1921, Schwab et al., 1959, Schwab and Zieper, 1965, Flowers, 1975, Hallet and Khosbin, 1980). More recently, diminished levels of motor energy in patients with PD have been attributed to their preferential selection of movements from the lower end of an underlying distribution of physiological capabilities (Mazzoni et al., 2007, Mazzoni et al., 2012). In the current study, it is equally plausible that despite instruction to execute maximal handgrips, our patients continued to ‘select’ submaximal responses from such a distribution. In this context, the arousing auditory stimuli delivered may have exerted their influence through an ‘energization’ of movement (Ballanger et al., 2006), resulting in an increased probability of selection of stronger and faster grip parameters (Anzak et al., 2011a, Anzak et al., 2011b).

Our results thus suggest that voluntary movements, though scaled down as a result of dopaminergic deficiency and basal ganglia dysfunction in PD (Anzak et al., 2011b), are still normally influenced by largely dopamine independent (see Results) pathways involved in the processing of phasic arousal, and thereby arousal induced energizing influences on movement.

The relationship between the subcortical potential identified in the present study and the cortical P50, also considered a marker of arousal, is unclear (Erwin and Buchwald, 1986, Miyazato et al., 1999, Reese et al., 1995, Skinner et al., 1999, Baruth et al., 2010). It too has been posited to originate from the PPN, although this is a subject of considerable debate (Garcia-Rill et al., 2011). The intensity of the auditory stimuli used in the current study was sufficient to induce eye blinks which precluded EEG recordings of a concurrent cortical response. In the future it would be interesting to simultaneously record subcortical and cortical mid-latency activities in response to lower intensity stimuli that do not elicit blink reflexes and to further test the reactivity and habituation of the evoked activities with stimuli presented at frequencies above 1 Hz (Miyazato et al., 1995). In addition, it would be interesting to derive a further direct measure of arousal during the presentation of imperative auditory cues of graded intensity.

To summarize, irrespective of the proposed influence of the PPN, our results suggest that evoked activity in the STNr may contribute to enhancements in force over and above maximal effort of will. Thus a strong correlation between the amplitude of the mid-latency evoked potentials in the STNr and facilitation of peak force was observed. There were also significant correlations between the evoked potential and peak yank increments and reaction time decrements, albeit weaker than those with peak force.

Two general limitations of this study should be highlighted though. First, circumspection is warranted in interpreting the potential physiological associations and functions of the mid-latency evoked potential identified here, given that data were necessarily recorded in patients with PD. That said, motor performance in response to an intense auditory stimulus is similarly improved following loud auditory cues in both patients with PD and healthy age-matched controls (Anzak et al., 2011a and present results). Second, our findings remain correlative in nature and therefore cannot be taken as proof of a causal link between the evoked activity in the STN and PPN, and improvements in motor performance. Interestingly, however, the shortening of reaction time following loud stimuli is diminished in Parkinsonian patients with prominent gait freezing, which has been associated with pathological abnormalities in the PPNr (Thevathasan et al., 2011, Nonnekes et al., 2014). Moreover, therapeutic stimulation of the PPN can reverse this impairment (Thevathasan et al., 2011, Nonnekes et al., 2014).

## 5 Conclusions

In conclusion, our results identify an evoked potential locally generated in the STNr, the amplitude of which scales with both auditory cue intensity and enhancements in motor performance over and above maximal effort of will. We further provide evidence of an evoked potential with similar latency and reactivity to cue intensity but with focal origin in the PPNr, a key component of the RAS. In sum, our findings suggest that the subcortical mid-latency evoked potential identified may be a correlate of arousal related energization of voluntary movement. Manipulation of this system may provide a novel approach for the non-dopaminergic enhancement of motor performance in patients with hypokinetic disorders such as PD.

## Figures and Tables

**Table 1:** Patient details. Surgical sites: (1) National Hospital for Neurology & Neurosurgery, London, (2) John Radcliffe Hospital, Oxford, (3) Kings College Hospital, London, (4) Frenchay Hospital, Bristol. UPDRS scores for Patient 8 were not available.

Site	Bilateral targets	Patient no.	Age/yrs	Disease duration/yrs	Daily L-DOPA equivalent dose/mg	Pre-op UPDRS OFF/ON Levodopa
1	STN	1	59	15	700	28/5
1	STN	2	60	17	1725	63/7
1	STN	3	32	10	875	52/13
1	STN	4	56	10	400	40/12
2	STN	5	70	12	1100	62/29
2	STN	6	60	7	200	25/13
3	STN	7	56	10	900	26/7
3	STN	8	64	12	300	n/a
4	STN& PPN	9	68	12	475	38/20

**Table 2:** Results of repeated measures ANOVAs with factors (1) stimulus intensity (82, 88, 94, 100 and 105 dB) and (2) medication state (OFF and ON dopaminergic medication), applied to peak force and reaction time data. n = 16 gripping hands. Significant values are emboldened. The greater peak forces attained by subjects in OFF medication compared to those in ON likely reflect a fatigue effect in ON drug recordings which wereundertaken secondly.

	Peak force	Reaction time
Stimulus intensity	F_4,60_ = 3.369 **p** **=** 0.015*	F_4,60_ = 4.487 **p** **=** 0.003*
Medication state	F_1,15_ = 5.357 **p** **=** 0.035* (OFF > ON L-DOPA)	F_1,15_ = 0.004 p = 0.951
Stimulus intensity ∗ medication state	F_4,60_ = 0.584 p = 0.675	F_4,60_ = 0.382 p = 0.821

**Table 3:** Results of separate repeated measures ANOVAs for OFF and ON L-DOPA recordings applied to each individual's mean peak evoked potential amplitudes, with factors (1) stimulus intensity (82, 88, 94, 100 and 105 dB) and (2) hemisphere (left versus right STNr) (n = 16 experimental runs). Significant values are emboldened.

	OFF L-DOPA	ON L-DOPA
Stimulus intensity	F_4,60_ = 7.365 **p** **=** 0.001*	F_4,60_ = 5.766 **p** **=** 0.003*
STNr side	F_1,15_ = 0.441 p = 0.517	F_1,15_ = 0.002 p = 0.963
Stimulus intensity ∗ STNr side	F_4,60_ = 0.660 p = 0.513	F_4,60_ = 1.484 p = 0.218
