# Supplementary material for: Subcortical evoked activity and motor enhancement in Parkinson's disease
Source: Exp Neurol. 2016 Mar;277:19–26. doi: 10.1016/j.expneurol.2015.12.004 (PMC4767325; doi:10.1016/j.expneurol.2015.12.004)

**Supplementary Material 1: Derivation of evoked activity and event-related induced LFP power**

Evoked activity was derived from bilateral DBS electrodes by averaging across trials. The *absolute* peak amplitudes of evoked potentials (lying between 50-100ms; Baruth et al, 2010), were derived as an average over a 10ms window centered around the peak. Induced power was derived by subtracting the average across trials from the original local field potential measurement of each trial to avoid contamination from evoked potentials. A time-frequency decomposition based on the continuous wavelet transform was then applied to each (average-subtracted) trial to analyze changes in induced LFP activity in the time-frequency domain. The wavelet function was convolved with the observed data at each time point across the range of frequencies, allowing the identification of specific frequency components over time. In the present study, we used the Morlet wavelet function where a sinusoidal oscillation () is modulated by a Gaussian bell curve () to give a brief oscillation localized in both time and frequency (Weinberger et al, 2009). The absolute value of the transform was squared to give a continuous time-frequency representation of the power content of the signal.

Event-related induced LFP power was subsequently calculated by normalizing the power at each time point against the average power between two seconds and one second before the cue, so that a value higher than zero indicated power higher than before cue and *vice versa*. The normalized power induced at different frequencies and at different time points was aligned to cue presentation, and averaged across trials of a given type and subsequently across the three bipolar contacts for each STN electrode contralateral to the gripping hand. Averages across all the contact pairs in a given electrode were calculated, so as to avoid selection bias. The resultant time-evolving power spectra of changes in STNr LFPs induced by cues at each intensity, relative to a pre-cue baseline, enabled five frequency bands to be identified over which increases or decreases in power (in the time period from cue onset to response onset) were distinguishable from the pre-cue baseline and from neighbouring bands. The five frequency ranges identified as responsive to the imperative auditory-visual cues, common to all experimental conditions, were thus: theta/alpha (5-12Hz), low beta (13-19 Hz), intermediate beta (20-25 Hz), high beta (26-33 Hz), and broad gamma (34-375 Hz). As a data-driven approach - specific to the power spectra derived from this study - was adopted, the precise definitions of the reactive frequency bands differ slightly from that in our previous work (Anzak et al, 2012). Most notable is the presence of synchrony over a narrow intermediate beta band which is more pronounced in OFF L-DOPA recordings (see also Kempf et al, 2007), and a high beta desynchrony that extends to marginally higher frequencies than previously described.

***Supplementary References***

Baruth JM, Casanova MF, Sears L, Sokhadze E. Early-stage visual processing abnormalities in high-functioning autism spectrum disorder. Transl Neurosci. 2010; 1:177-87.

Kempf F, Kühn AA, Kupsch A, Brücke C, Weise L, Schneider GH et al. Premovement activities in the subthalamic area of patients with Parkinson's disease and their dependence on task. Eur J Neurosci. 2007;25:3137-45.

Weinberger M, Hutchison WD, Lozano AM, Hodaie M, Dostrovsky JO. Increased gamma oscillatory activity in the subthalamic nucleus during tremor in Parkinson's disease patients. J Neurophysiol 2009;101:789-802.

**Supplementary Material 2: Results pertaining to Peak Yank**

***Progressive enhancements in PY with increasing stimulus intensity***

Like PF, PY increased with stimulus intensity, with the exception that the responses to the highest two and lowest two sound intensities were indistinguishable (**Supplementary Figure 2ai**). A repeated measures ANOVA confirmed significant effects of stimulus intensity on PY (F_4,60_=3.643, p=0.010), but no effect of dopaminergic medication (F_1,15_=1.431, p=0.250) nor stimulus x medication interaction (F_4,60_ =0.367, p=0.831). Group average PY across dopaminergic states changed from 148.6 ± 18.0 kg/s in response to the lowest intensity stimulus to 163.4 ± 17.8 kg/s in response to the highest intensity stimulus. In healthy subjects too, PY increased with stimulus intensity (**Supplementary Figure 2aii**). A repeated measures ANOVA confirmed significant effects of stimulus intensity on PY (F_4,44_=20.802, p<0.001), but no effect of experimental run (F_1,11_=1.2.437, p=0.147) nor stimulus x experimental run (F_4,44_ =1.535, p=0.236). Group average PY across experimental runs changed from 96.0 ± 17.0 kg/s in response to the lowest intensity stimulus to 124.0 ± 20.5 kg/s in response to the highest intensity stimulus. When averaging across experimental runs and comparing to the mean results of the PD group, a repeated measures ANOVA identified significant effects of stimulus intensity on PY (F_4,44_=13.117, p<0.001), no effect of experimental run/drug state (F_1,11_=1.700, p=0.219), but did suggest a stimulus x experimental run/drug state interaction (F_4,44_ =2.772, p=0.039).

***Enhancements in PY correlate with amplitude of the STNr short latency evoked potential***

PY (from 16 gripping hands) and peak amplitudes of the 50-100ms STNr evoked potential were normalized to the average responses to the lowest stimulus intensity, so that relationships could be investigated at the within-subject level. Simple regression analysis (applied to log transformed data) identified a significant relationship between the peak amplitude of the STNr evoked potential and PY (r=0.477, p<0.001) **(Supplementary Figure 2b)**. However, as with PF, PY did not correlate with stimulus intensity in a simple linear manner (r=0.217, p=0.086). A multiple regression model that included both peak evoked potential amplitude and transformed mean LFP power of induced components over the five identified frequency bands (ie. six predictive variables in total) revealed that only evoked potential amplitude, and not induced frequency-specific activities, contributed to PY (β = 0.488, p < 0.001*). The model fit was good: F=4.395, p=0.001, R^2^ = 0.332.

|  | **Peak Force** | **Reaction Time** |
| --- | --- | --- |
| **Stimulus intensity** | F_4,44_= 19.144  **p<0.001 *** | F_4,44_= 18.196  **p<0.001** * |
| **Experimental run** | F_1,11_= 0.656  p=0.453 | F_1,11_= 3.838  p=0.076 |
| **Stimulus intensity x**  **Experimental run** | F_4,44_= 1.958  p=0.118 | F_4,44_= 1.700  p=0.177 |

**Supplementary Table 1**. Repeated measures ANOVAs applied to mean Peak Force and Reaction Times in healthy subjects (n=12 gripping hands). Factors included: stimulus intensity (5 different levels) and experimental run (first vs second).

|  | **Peak Force** | **Reaction Time** |
| --- | --- | --- |
| **Stimulus intensity** | F_4,44_= 11.913  **p<0.001 *** | F_4,44_= 4.669  **p=0.027 *** |
| **Group** | F_1,11_= 1.349  p=0.270 | F_1,11_= 10.443  **p=0.008 *** |
| **Stimulus intensity x**  **Group** | F_4,44_= 1.679  p=0.172 | F_4,44_= 0.233  p=0.760 |

**Supplementary Table 2.** Repeated measures ANOVAs with factors Group (patients vs healthy subjects) and stimulus intensity (5 different levels).

|  | **Stimulus intensity** | **Medication status** | **Stimulus intensity ***  **medication status** |
| --- | --- | --- | --- |
| 5-12 Hz  Theta/Alpha | F_4,60_= 0.266, p=0.755 | F_1,15_= 0.889, p=0.361 | F_4,60_= 0.764, p=0.492 |
| 13-19 Hz  Low Beta | F_4,60_= 0.807, p=0.432 | F_1,15_= 0.670, p=0.426 | F_4,60_= 1.157, p=0.332 |
| 20-25 Hz  Intermediate Beta | F_4,60_= 1.405, p=0.261 | F_1,15_= 5.659, **p=0.031*** | F_4,60_= 1.157, p=0.339 |
| 26-33 Hz  High Beta | F_4,60_= 1.557, p=0.197 | F_1,15_= 3.720, p=0.073 | F_4,60_= 2.022, p=0.103 |
| 34-375 Hz  Broad Gamma | F_4,60_= 1.049, p=0.348 | F_1,15_= 0.010, p=0.922 | F_4,60_= 2.160, p=0.151 |

**Supplementary Table 3.** Repeated measures ANOVAs applied to transformed mean induced LFP power derived from the time period between cue onset and time to movement onset, separately for the five reactive frequency bands identified. Factors included: stimulus intensity (5 different levels) and medication status (OFF vs ON L-DOPA). The time period analyzed was selected to minimize the inclusion of activities related to peripheral afferance during the movement phase. In this time period, intermediate beta was found to be more synchronized, relative to the pre-cue baseline, in the ON L-DOPA as compared to OFF L-DOPA state. **Supplementary Figure 6,** however, suggests a reversal of this effect once the grip had been initiated. No significant effect of cue intensity was identified.


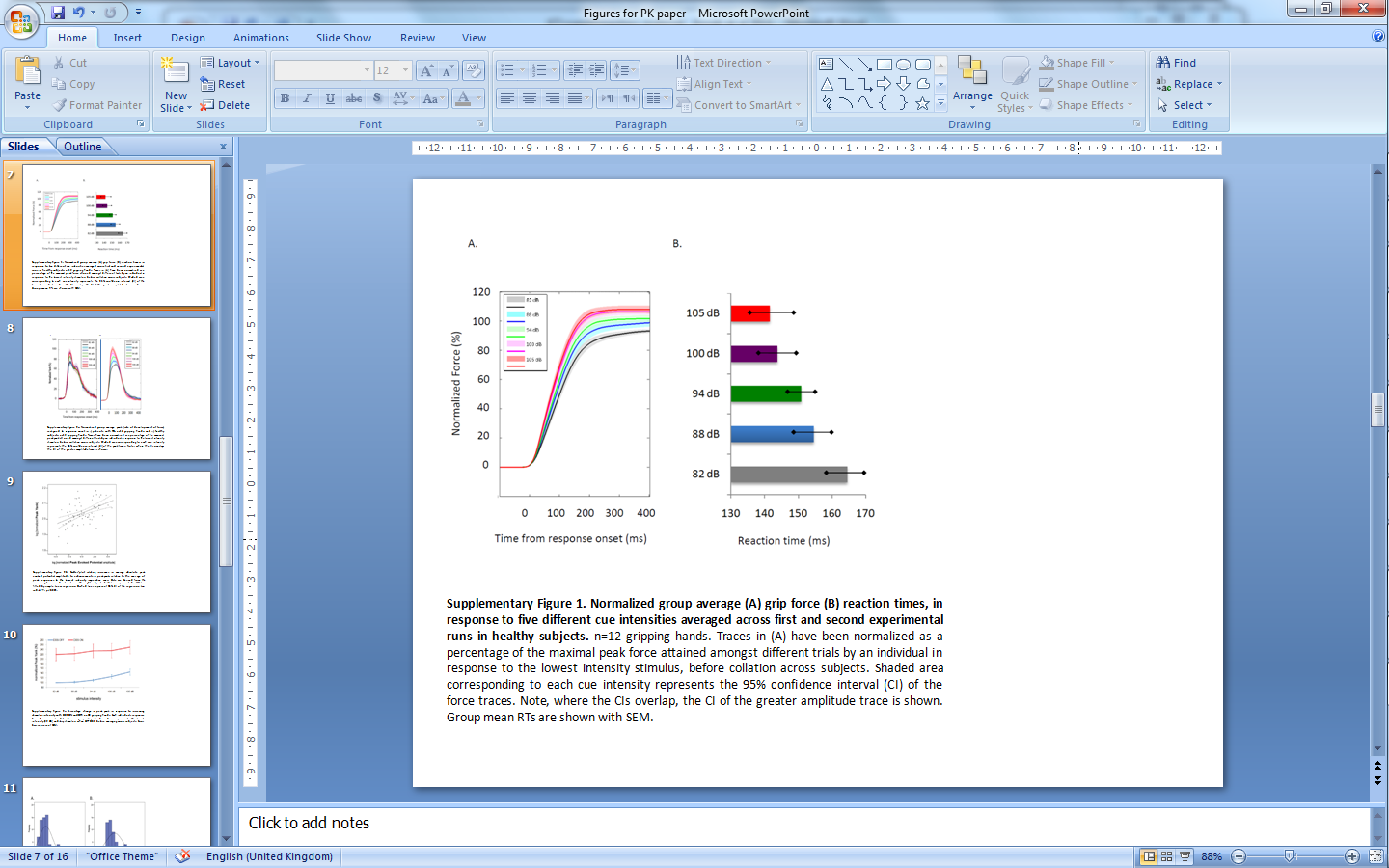


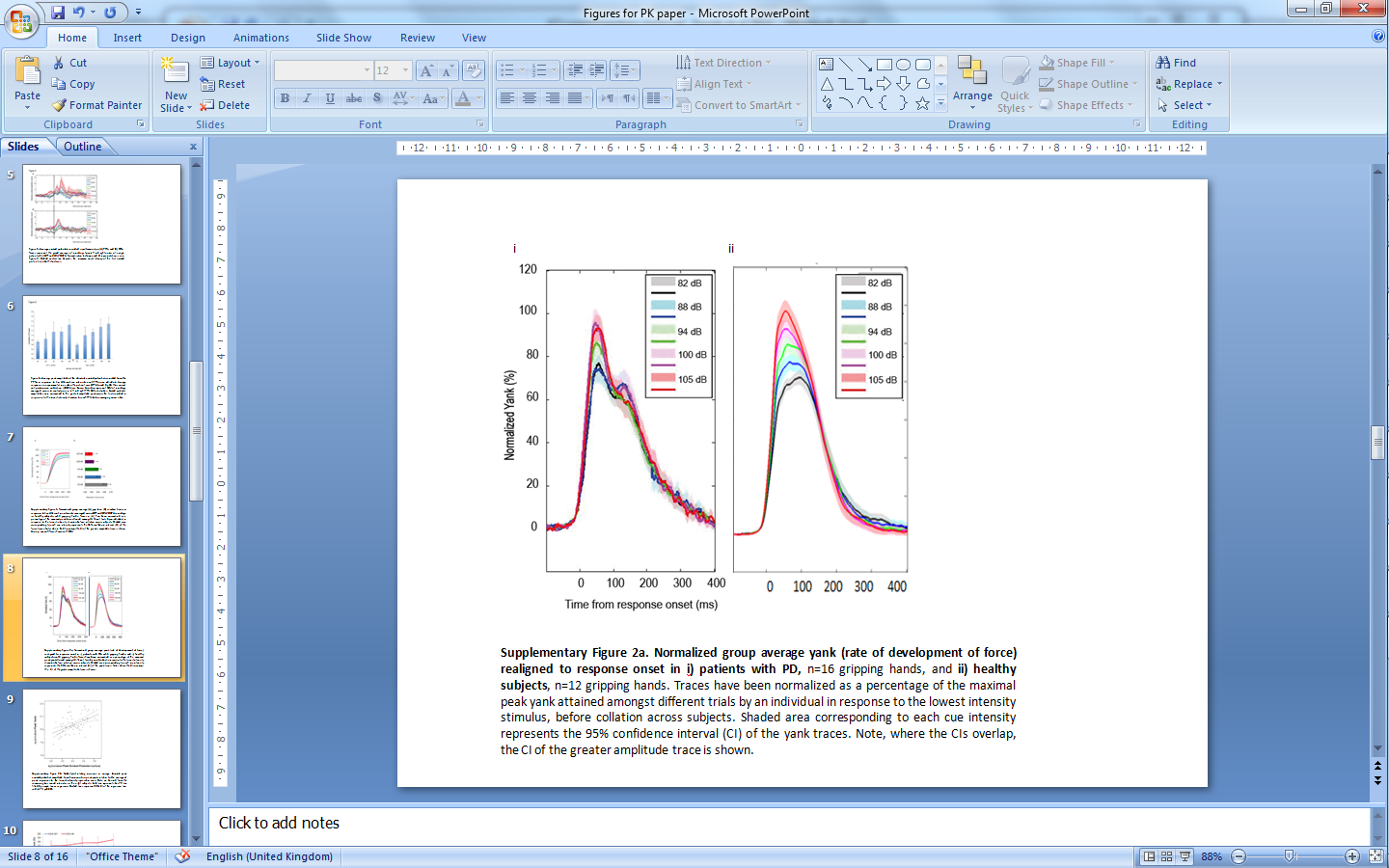


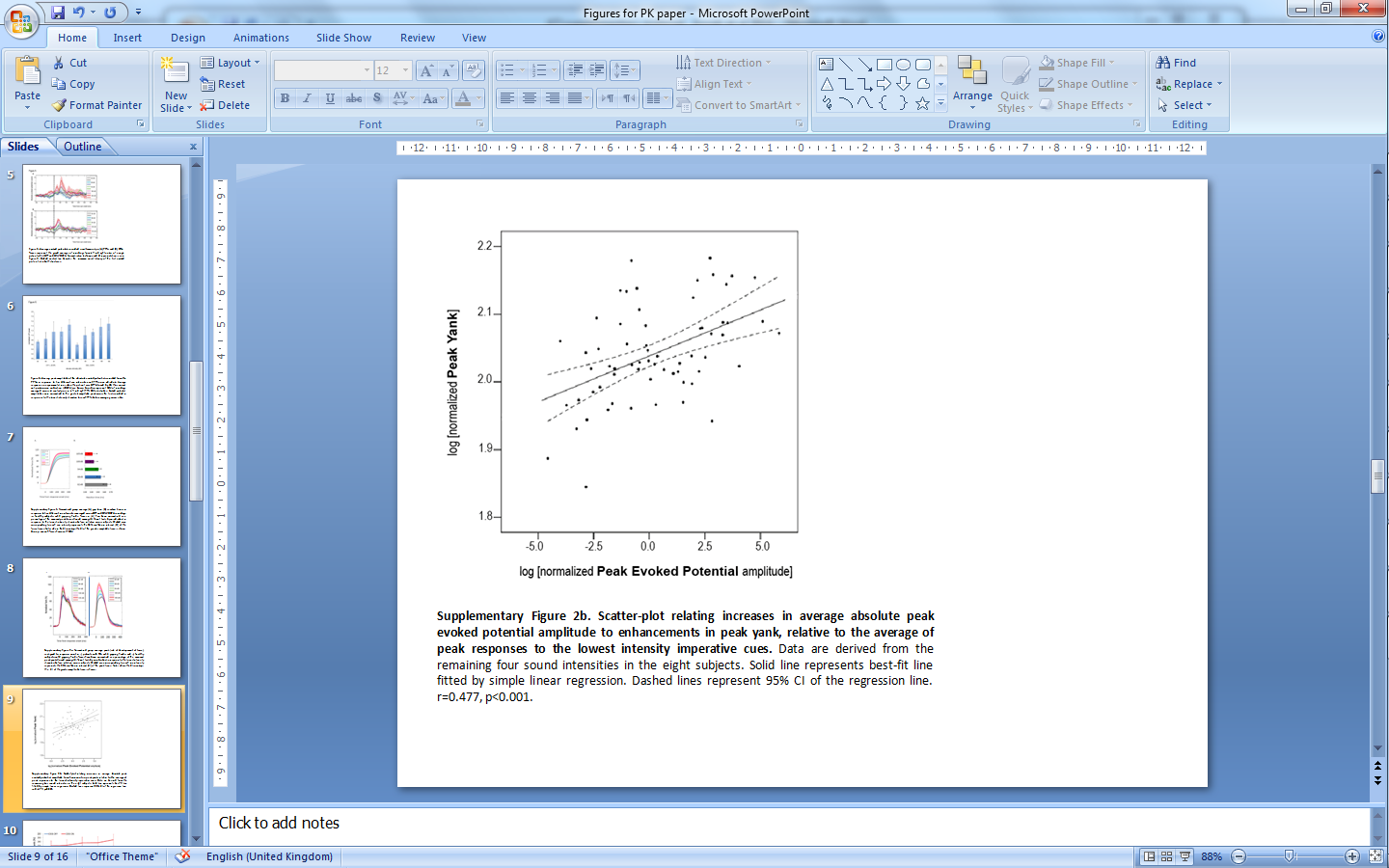


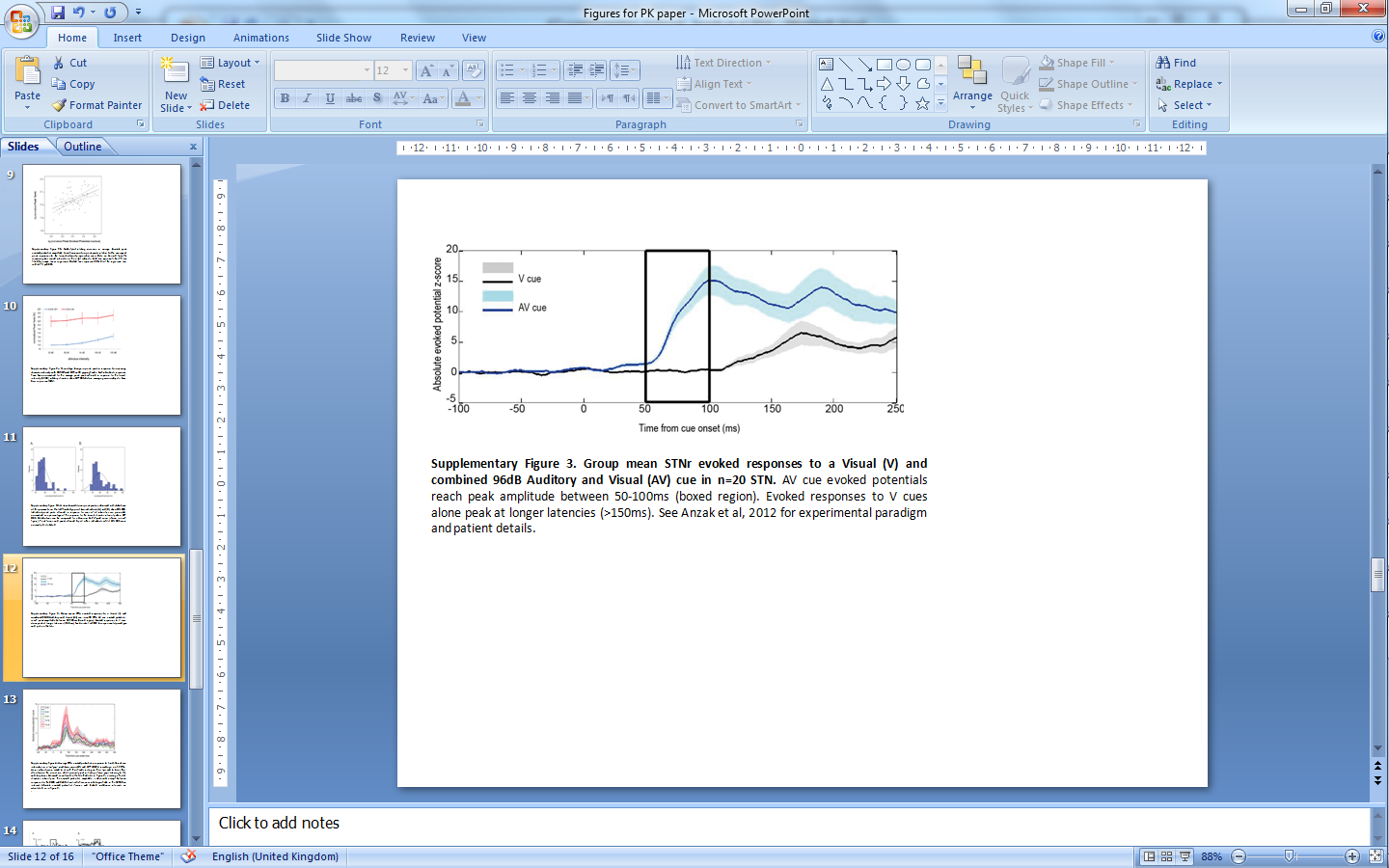


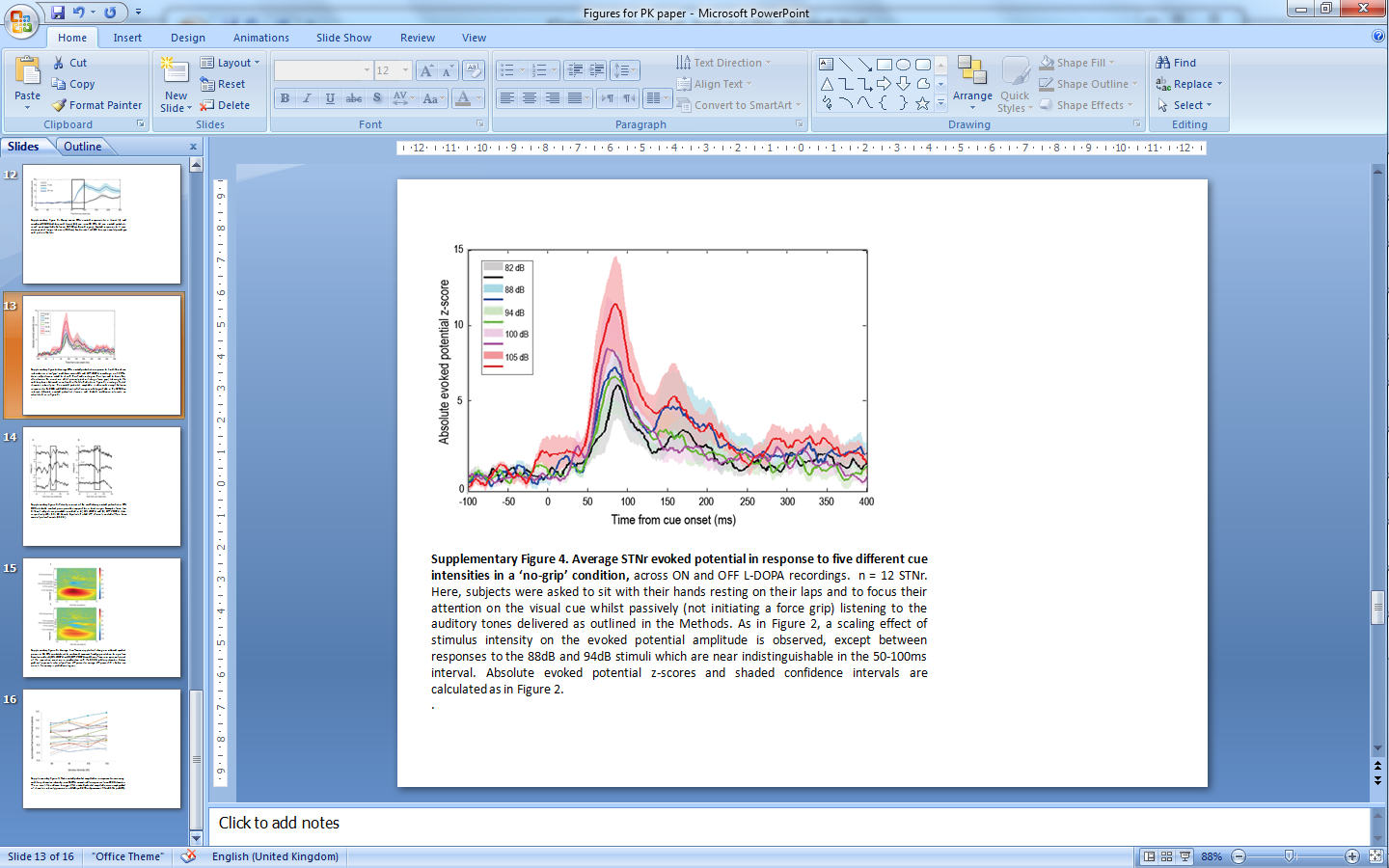


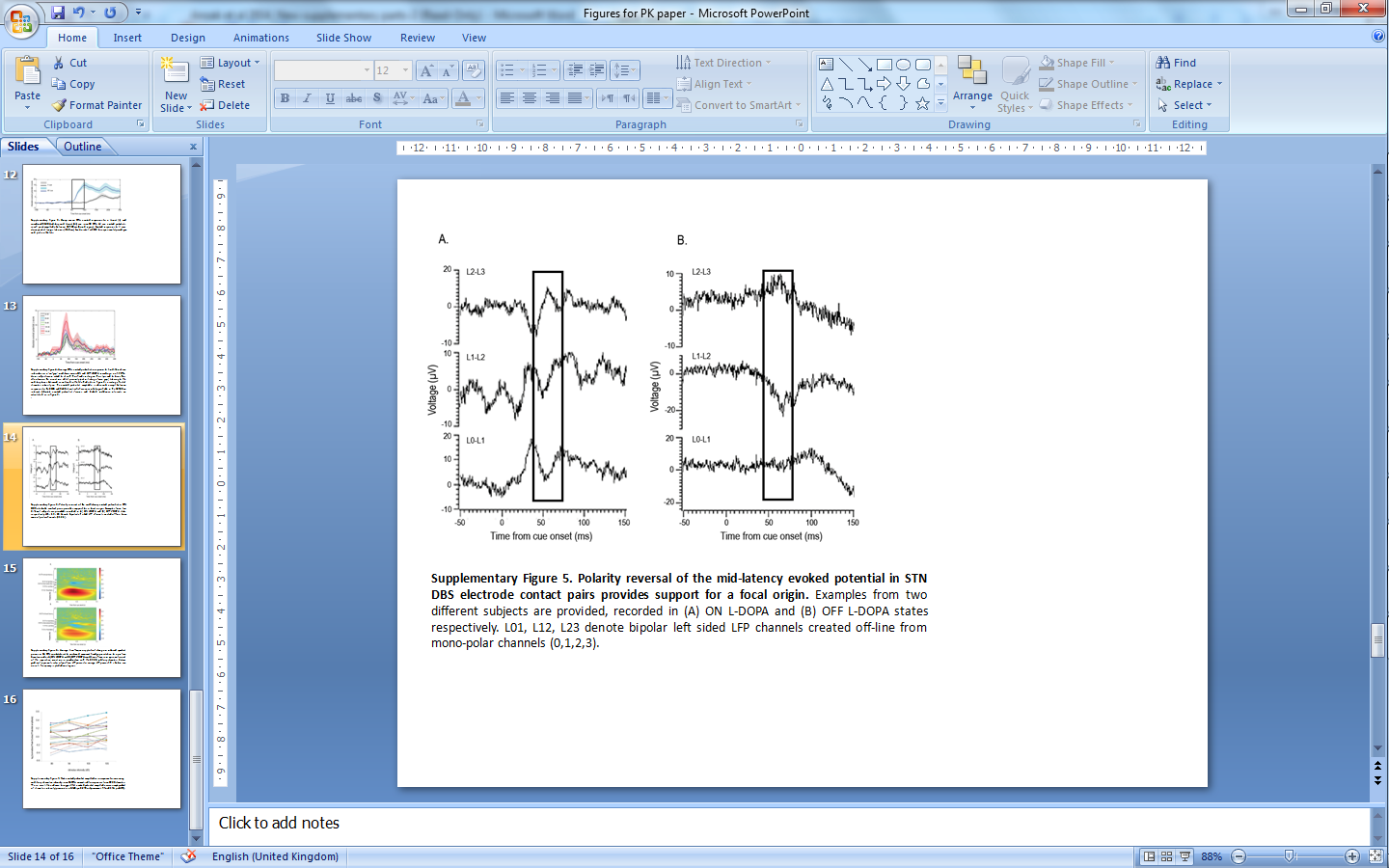


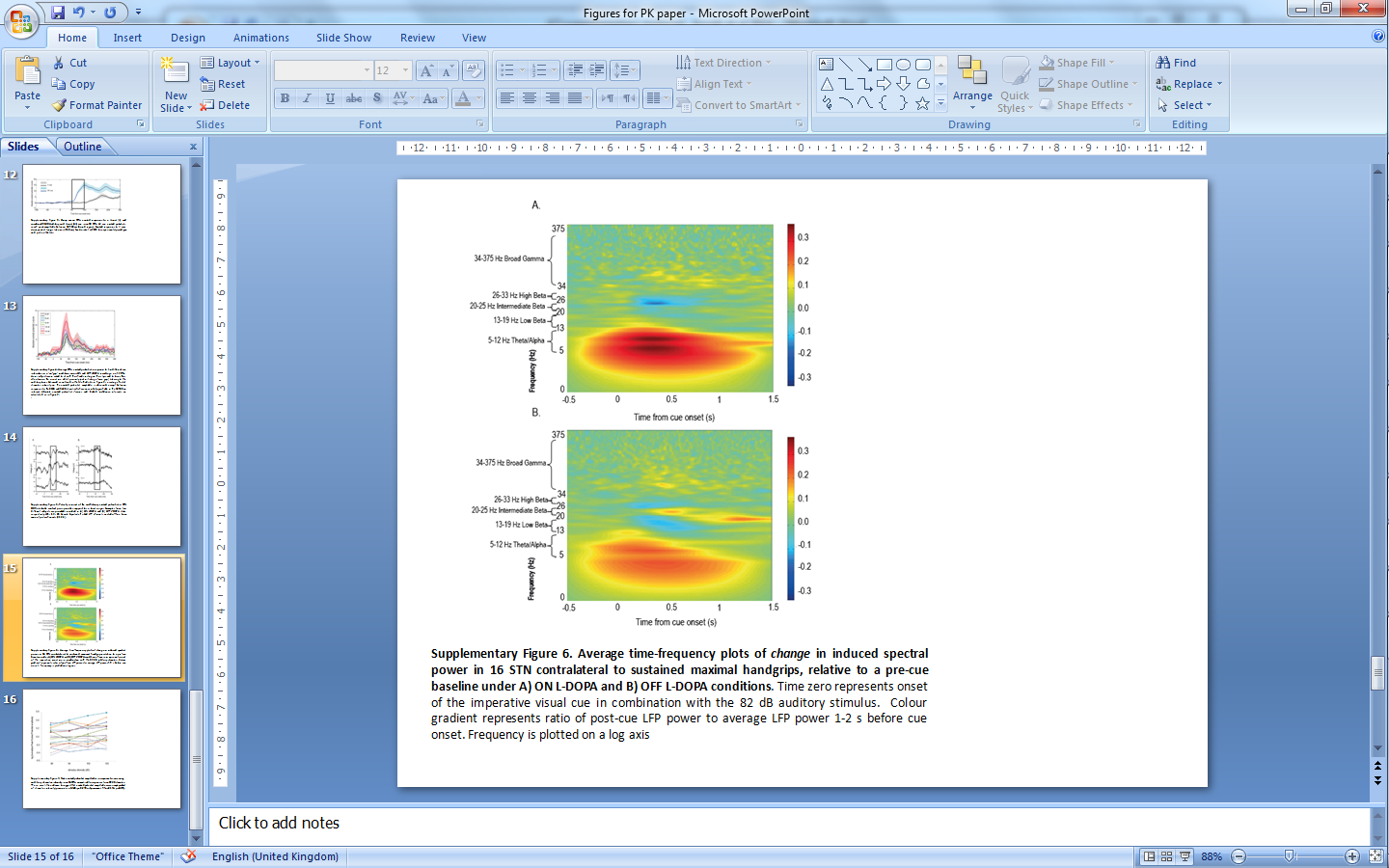


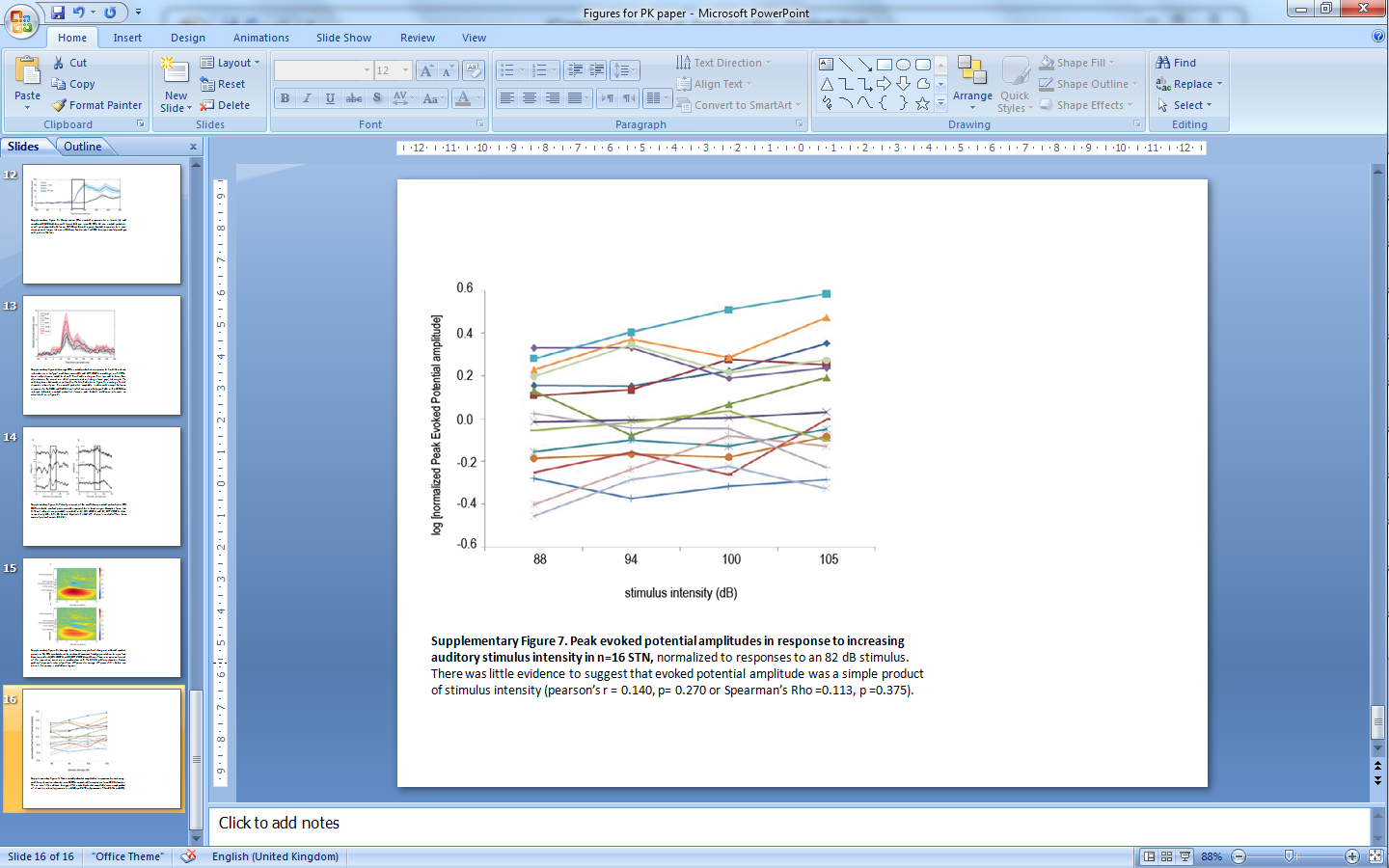

Supplement: Supplementary file 1 — Supplementary material [file mmc1.docx]
